# Morphological Differences in *Pinus strobiformis* Across Latitudinal and Elevational Gradients

**DOI:** 10.3389/fpls.2020.559697

**Published:** 2020-10-22

**Authors:** Alejandro Leal-Sáenz, Kristen M. Waring, Mitra Menon, Samuel A. Cushman, Andrew Eckert, Lluvia Flores-Rentería, José Ciro Hernández-Díaz, Carlos Antonio López-Sánchez, José Hugo Martínez-Guerrero, Christian Wehenkel

**Affiliations:** ^1^Programa Institucional de Doctorado en Ciencias Agropecuarias y Forestales, Universidad Juárez del Estado de Durango, Durango, Mexico; ^2^School of Forestry, Northern Arizona University, Flagstaff, AZ, United States; ^3^Department of Evolution and Ecology, University of California, Davis, Davis, CA, United States; ^4^USDA Forest Service, Flagstaff, AZ, United States; ^5^Department of Biology, Virginia Commonwealth University, Richmond, VA, United States; ^6^Department of Biology, San Diego State University, San Diego, CA, United States; ^7^Instituto de Silvicultura e Industria de la Madera, Universidad Juárez del Estado de Durango, Durango, Mexico; ^8^Department of Biology of Organisms and Systems, Mieres Polytechnic School, University of Oviedo, Campus Universitario de Mieres, C/Gonzalo Gutiérrez Quirós S/N, Mieres, Spain; ^9^Facultad de Medicina Veterinaria y Zootecnia, Universidad Juárez del Estado de Durango, Durango, Mexico

**Keywords:** phenotypic variation, morphological traits, climate factors, redundancy analysis, multivariate canonical ordination, machine learning

## Abstract

The phenotype of trees is determined by the relationships and interactions among genetic and environmental influences. Understanding the patterns and processes that are responsible for phenotypic variation is facilitated by studying the relationships between phenotype and the environment among many individuals across broad ecological and climatic gradients. We used *Pinus strobiformis*, which has a wide latitudinal distribution, as a model species to: (a) estimate the relative importance of different environmental factors in predicting these morphological traits and (b) characterize the spatial patterns of standing phenotypic variation of cone and seed traits across the species’ range. A large portion of the total variation in morphological characteristics was explained by ecological, climatic and geographical variables (54.7% collectively). The three climate, vegetation and geographical variable groups, each had similar total ability to explain morphological variation (43.4%, 43.8%, 51.5%, respectively), while the topographical variable group had somewhat lower total explanatory power (36.9%). The largest component of explained variance (33.6%) was the four-way interaction of all variable sets, suggesting that there is strong covariation in environmental, climate and geographical variables in their relationship to morphological traits of southwest white pine across its range. The regression results showed that populations in more humid and warmer climates expressed greater cone length and seed size. This may in part facilitate populations of *P. strobiformis* in warmer and wetter portions of its range growing in dense, shady forest stands, because larger seeds provide greater resources to germinants at the time of germination. Our models provide accurate predictions of morphological traits and important insights regarding the factors that contribute to their expression. Our results indicate that managers should be conservative during reforestation efforts to ensure match between ecotypic variation in seed source populations. However, we also note that given projected large range shift due to climate change, managers will have to balance the match between current ecotypic variation and expected range shift and changes in local adaptive optima under future climate conditions.

## Introduction

The interest of evolutionary ecologists has long been focused on patterns of phenotypic variation along environmental gradients (e.g., latitude, altitude and climate). However, this variation is also determined by the covariance between genetic and environmental influences (Conover et al., 2009). Studying patterns of phenotypic variation among many individuals along broad ecological and climatic gradients is a powerful framework to understand the patterns and processes that govern phenotypic expression and variation (Endler, 1986). Early ecologists frequently noted that phenotypes of many species change predictably along large-scale gradients of latitude, altitude and water depth, providing the basis for several so-called ecological rules (Bergmann, 1847; Atkinson and Sibly, 1997). Morphological traits are important characteristics (Violle et al., 2007) that help distinguish entities at all levels of biological organization, including life cycles, ecological and geographical distributions, and evolutionary and conservation status (Kaplan, 2001; Gregorius et al., 2003). For example, Delagrange et al. (2004) showed that the crown morphology of *Acer saccharum* and *Betula alleghaniensis* is influenced by light availability and tree size. Wahid et al. (2006) found that the morphology of *Pinus pinaster* cones and needles vary predictably along both latitudinal and altitudinal gradients and Gil et al. (2002) reported elevation-dependent cone and seed variation in *Pinus canariensis*.

Forest trees are often adapted to environmental gradients at multiple spatial scales (Morgenstern, 1996; Savolainen et al., 2007). The phenotype of an individual is influenced by their genotype, their environment and interactions between them (de Jong, 1990; Falconer and Mackay, 1996). The evolutionary response of a phenotypic trait to selection depends on genetic control of the trait, heritability of the trait and differential fitness of different morphotypes of the trait in different environmental conditions (Price, 1970). Quantifying morphological variation within a widely distributed species across a large spatial extent is often necessary to identify the relative importance of different environmental factors in relation to variation in intraspecific morphological traits (Boyd, 2002; Herrera et al., 2002; Franks et al., 2014; Ji et al., 2016). Such knowledge can then be applied to climate change adaptation, through strategies such as assisted migration and assisted gene flow (e.g., Aitken and Bemmels, 2016).

**FIGURE 1:**
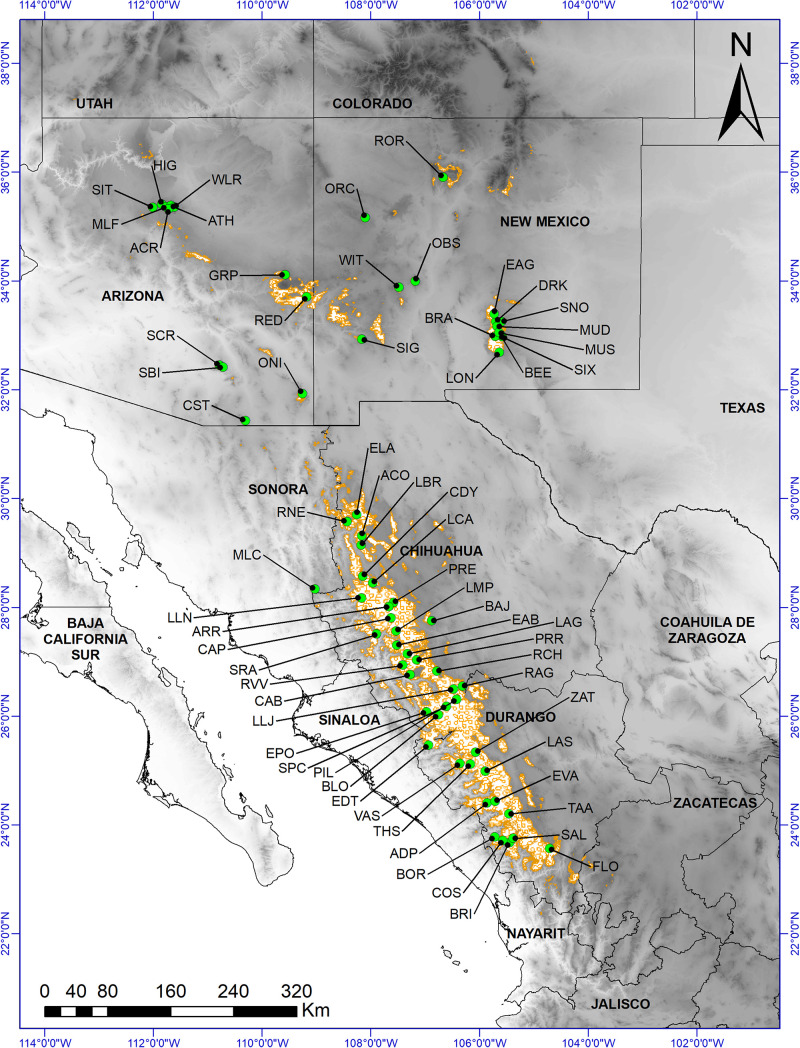
Distribution of *Pinus strobiformis* (brown outlined areas, based on Shirk et al., 2018) and sample collection sites: 65 morphological data collection sites (green circles). Base digital elevation map was from Jarvis et al. (2008).

White pine species (subgenus *Strobus*, section *Quinquefoliae*, subsection *Strobus* (Gernandt et al., 2005)) are widespread in the temperate forest ecosystems of North America (Kral, 1993; Gernandt and Pérez-de la Rosa, 2014), making them a suitable focal group to investigate how the phenotype is influenced by environmental gradients. Members of subgenus *Strobus*, section *Quinquefoliae*, subsection *Strobus* have five needles per bundle, such that species in this group are commonly referred to as five-needle pines. Several white pines are also characterized by large cones (Farjon and Styles, 1997) and large seeds (Frankis, 2009). These include the southwestern white pine (*Pinus strobiformis* Engelm.), which has wide variation in cone length, 20–50 cm (Frankis, 2009), seed length, 1.2–1.8 cm (Farjon and Styles, 1997), seed weight of 0.140–0.411 g (Leal-Sáenz et al., 2020), and a geographical range that includes the Sierra Madre Occidental in Mexico and the southwestern United States (Figure 1). Southwestern white pine is of both commercial and ecological value. Commercial products include timber, cellulose, resin and pulp (Farjon and Styles, 1997; Villalobos-Arámbula et al., 2014).

Ecologically, *P. strobiformis* is an important tree species in the ecosystems it inhabits (Moreno-Letelier and Piñero, 2009; Bower et al., 2011; Looney and Waring, 2013). The large and nutritious seeds of white pines frequently form the foundation of large trophic networks and promote enhanced biodiversity (Keane and Cushman, 2018). Seeds are an important source of food for many birds and mammals, including corvids, parrots, mice, voles, chipmunks, squirrels and bears (Samano and Tomback, 2003; Tomback and Achuff, 2010; Looney and Waring, 2013). In addition to contributing to biodiversity, *P. strobiformis* also provides watershed protection and serves as a major floristic component of montane forest ecosystems (Wehenkel et al., 2014), and is a key element of mixed conifer forests across its range (Looney and Waring, 2013; Shirk et al., 2018).

*Pinus strobiformis* is highly susceptible to white pine blister rust (WPBR), an invasive tree disease caused by the fungus *Cronartium ribicola* (Geils and Vogler, 2011), as are all other white pine species (Hoff and Hagle, 1990; Sniezko et al., 2008). The dual threats of this non-native fungal pathogen and the projected warmer, drier climate have created an uncertain future for *P. strobiformis*, as well as other white pine species (Landguth et al., 2017; Keane and Cushman, 2018; Shirk et al., 2018). A contraction of more than 60% in the species range by 2080 is predicted under some scenarios of greenhouse gas emissions (Shirk et al., 2018), whereas other scenarios include a northerly shift of more than 1,000 km in the mean latitude and an increase of 500 m in the mean elevation of suitable habitat for the species.

Robust measurement of the degree to which morphological variation is determined by non-genetically controlled developmental responses to environmental gradients requires a statistical analysis that can jointly and simultaneously account for the ability of environmental factors to predict morphological characteristics. RDA is a multivariate direct ordination approach that is ideally suited to predicting multiple response variables as a function of multiple predictor variables and is the most well-known and trusted statistical method to conduct multivariate variance partitioning (Van Den Wollenberg, 1977; McGarigal et al., 2000). By implementing a series of partial redundancy analysis ordinations, the independent and joint effects of multiple sets of predictor variables can be separated (e.g., Borcard et al., 1992; Cushman and McGarigal, 2002, 2004). For instance, the proportion of morphological trait variance explained by pure effects of climatic data, spatial location or topography and the interaction between variables can be quantified. A series of partial redundancy analyses can then be used to identify environmental gradients correlated with phenotypic variation across the species range. This partitioning of the variance in morphological traits that can be explained independently and jointly by climatic and geographic factors is essential to understand the factors that drive morphological variation and the scales at which they operate.

Faced with an increasingly complex and rapidly changing future, forest managers need science-driven strategies to maintain tree species and forests. Given the ecological importance of *Pinus strobiformis* and the multiple challenges to future regeneration of the species, it is critical to understand factors underlying the morphological trait variation of reproductive structures (e.g., cones and seeds) in order to better manage reforestation and restoration efforts in the future. We used extensive sampling across the full range of *Pinus strobiformis*, to (a) estimate the relative importance of the effects of different environmental factors on these morphological traits and (b) characterize the spatial patterns of standing phenotypic variation of cone and seed morphological traits.

## Materials and Methods

### Species and Study Sites

*Pinus strobiformis* is a widely distributed white pine, ranging from central and southern Arizona, New Mexico, western Texas, and southwestern Colorado in the United States, to the Sierra Madre Occidental in Mexico (Figure 1; Looney and Waring, 2013; Villagómez-Loza et al., 2014). According to the Mexican National Forest Inventory (2004–2009), performed by the Mexican National Forestry Commission (CONAFOR, 2009), *P. strobiformis* occurs within an area of about 2.3 million hectares in Mexico, mainly at elevations of between 2,500 and 3,000 m, although the suitable habitat is estimated to constitute a much larger area (Aguirre-Gutiérrez et al., 2015; but see Shirk et al., 2018). *P. strobiformis* occurs at elevations between 1,800 and 3,400 m in the southwestern United States (Looney and Waring, 2013) but occupies less than 2 % of the total forested area in Arizona (O’Brien, 2002).

In 2015, we collected cones and seeds from 65 *P. strobiformis* sites, located across a variety of abiotic conditions and a wide latitudinal gradient in Mexico and the United States and representing the current geographic distribution (Figure 1, Supplementary Tables S1–S5). Sites were selected based on accessibility and availability of ripe cones and seeds, with a minimum distance of 5.1 km between sites. All stands were situated in closed forests with minimal human disturbance (e.g., roads, cattle grazing, agriculture). Three to five trees with ripe cones were sampled at each site (a total of 297 trees). In each site, the sampled trees were separated by a minimum distance of 50 m, to minimize the chance of sampling the same genetic family.

### Tree, Cone and Seed Morphology Data Collection

For each tree we recorded: Latitude, longitude and elevation (m), diameter at breast height (cm), total tree height (m), first live branch height (m), and crown length (m, obtained from the difference between total height and first live branch height) (Supplementary Tables S1, S2).

A minimum of 15 ripe cones with no signs of the presence of insects or diseases were collected from each tree, stored and transported in labeled bags (total 4,455 cones). These cones were allowed to dry at ambient temperature and humidity until they opened, and the seeds were then extracted. Empty seeds were separated from filled seeds, either manually or with a blower. The filled seeds were then weighed in 10-seed lots (g) on a balance (González-Ávalos et al., 2006; Iglesias et al., 2012).

Ten ripe cones were selected at random from the 15 cones (or more) harvested from each tree, for measurements. Total length (cm) and width (cm) of the widest part of each cone were recorded (the width was measured with the angle of the cone facing upward and taking care to avoid exerting pressure on the cone) (Bramlett et al., 1977; González-Ávalos et al., 2006; Iglesias et al., 2012; Prieto-Ruíz et al., 2014). The cone angle was measured with a 360° circular protractor, by placing the upper stem of the cone aligned with 0° (Forde, 1964; Wheeler and Guries, 1982).

Three of the ten cones per tree were randomly chosen for further measurements; three scales of the upper and three of the lower half of each cone were selected and removed. These scales were chosen from each third of the cone circumference, at angles of approximately 0°, 120°, and 240°. A total of 2,673 scales from the upper and 2,673 from the lower part of the cones were gotten from the 297 trees. The length and width of each scale was then measured with a digital caliper (mm). The length of the scale to the tip and the width at the widest part of the scale were recorded. The angle of each scale was measured using a circular protractor and aligning each scale to 90°. Cone specific trait means were calculated for each tree and site.

### Environmental Factors and Hybrid Degree

For each site (Figure 1), the following abiotic and biotic factors were recorded to examine their impact on the morphological traits of *P. strobiformis*: geographical aspect (0° to 360°), slope (%), occurrence of *P. strobiformis* regeneration (presence), presence or not of *Ribes* spp. (an alternative host for the white pine blister rust pathogen) and presence or not of woody species in the neighborhood (Supplementary Tables S4, S5). We only recorded the presence of these species within a 20 m radius of each individual *P. strobiformis* tree and calculated the relative frequency of these species. The sampled trees were also examined for signs and symptoms of white pine blister rust, and the following rating system was applied when the disease was present: cankers present on branches (1), bole (2), or bole and branches (3) (Arvanitis et al., 1984); these ratings were then transformed into frequency of occurrence prior to analyses (Supplementary Table S2). The climate data (20 temperature and precipitation variables) were downloaded from the PRISM database for the period from 1961 to 1990^1^ (Supplementary Table S3). For further analysis, the geographical aspect (0° to 360°) was transformed into a cosine index – as 0° and 360° have the same zenithal aspect (Cushman and Wallin, 2002). Occurrence data were transformed into frequency of occurrence prior to analyses of the presence of trees and shrubs (Supplementary Tables S4, S5). We assumed that the presence of other tree species around *P. strobiformis* does not directly affect its morphological traits. However, this factor was used as additional proxy of other abiotic factors (such as soil traits) possibly influencing the morphological traits of *P. strobiformis* (Zhang et al., 2016).

Since *P. strobiformis* x *P. flexilis* hybrids, which were only reported in the US populations, could influence morphological traits, we also recorded the hybrid degree (the relative hybrid frequency or proportion of *P. strobiformis* to *P. flexilis* per stand) as predictor variable, following Menon et al. (2018) (Supplementary Table S1).

### Statistical Analyses

#### Detecting Spatial Dependence in Morphological Traits by Ordinary Kriging Analysis

We conducted ordinary kriging (ordinary Gaussian process regression model) to spatially interpolate morphological traits, including a 10-fold point-by-point cross validation (Batista et al., 2016). The statistical software R (version 3.3.4) (R Development Core Team, 2017) and the Interpolation Kriging package (ArcGIS Desktop 10.5, 2016) were used to describe first-order variation in the spatial pattern of the morphological cone and seed traits, as well as the dasometric traits of the *P. strobiformis* sampled trees under study. We tested the following mathematical models for the semivariance: the spherical model, exponential model, Gaussian model and the Stein’s parameterization. The corrected coefficient of determination between the observed and predicted values (*R*^2^), the Unbiased Root Mean Squared Error of the residuals (URMSE), the mean squared error and the mean absolute error were used to assess the goodness-of-fit. Finally, the cone and seed traits with the best kriging model were selected for further regression models of morphological traits with respect to environmental variables. The modeling was carried out using the “*SP*” (Pebesma and Bivand, 2005) and “*automap*” packages (Hiemstra et al., 2009) including the *CRS, SpatialPixelsDataFrame*, *autoKrige, autoKrige.cv*, and *compare.cv* functions in R (version 3.3.4) (R Development Core Team, 2017).

### Redundancy Analysis and Variance Partitioning

We implemented redundancy analysis with a multi-tiered variance partitioning method (e.g., Cushman and McGarigal, 2002), in the “*vegan*” R package (version 3.3.4) (Oksanen et al., 2010) to quantify the independent and joint ability of each set of environmental variables to predict the morphological characteristics of all sampled white pine individuals, and to measure the importance and joint interactive effects of the variables together. Importantly, this also enabled us to quantify the amount of morphological variation not explained by environmental variation in our data set. We computed a four-way partitioning among all climatic, vegetation, geographical and topographical variables. The spatial variables included the eigth spatial trend surface analysis terms (e.g., x, y, x^2, y^2, xy, x^2y, xy^2, and x^2y^2; Cushman and McGarigal, 2002).

### Selection of Independent Environmental Factors Influencing Morphological Traits

Selection of appropriate independent variables is fundamental for achieving effective predictive models (Krzanowski, 1987; Gnanadesikan et al., 1995; Maronna et al., 2018). We tested for significant differences in the mean values of explanatory environmental variables across the 65 *P. strobiformis* stands with morphological data (Supplementary Tables S1–S5) to describe variation across populations. Many of our predictor variables were not normally distributed (the variables are listed in Supplementary Tables S1–S5). Due to the absence of normality, we used the non-parametric Kruskal-Wallis test (Kruskal and Wallis, 1952) to evaluate whether the observed median’s differences in independent variables between *P. strobiformis* populations were statistically significant. All environmental variables for which we detected significant differences in median values of morphological traits (*α* = 0.01) were included in further analysis.

Additionally, the most important environmental factors influencing morphological variation were also determined using partial least squares and area under the univariate receiver operator curve, using the “varImp” function applied to the results of univariate analysis with the machine learning algorithm, using Random Forest [“caret” package, function train, methods “rf,” implemented in R (version 3.3.4; R Development Core Team, 2017)]. In each case, the unsupervised correlation filter was applied to the predictors prior to modeling (see details in the section “Regression techniques”).

The variable-importance measure was determined using partial least squares (Kuhn, 2012), on the basis of the weighted sums of the absolute regression coefficients. The weights are a function of the reduction of the sums of squares across the number of partial least squares components and are computed separately for each outcome. The contributions of the coefficients are thus weighted proportionally to the reduction in the sums of squares. The trapezoidal rule was used to compute the area under the receiver operator curve, which was used as a measure of the variables importance (Kuhn, 2012).

Finally, we used the non-parametric Spearman’s coefficient (*r*_*s*_) to determine correlations between the variables and to estimate collinearity between important independent variables (selected by the Kruskal-Wallis test, partial least squares or receiver operator curve). When the *r*_*s*_ absolute value for the difference between two variables was greater than 0.70, only the variable with the lowest *p* value in the Kruskal–Wallis test was included in the regression models (as reported by Salas et al., 2017 and Shirk et al., 2018). The relationships between the most important variables and two most spatially dependent cone and seed traits were represented graphically.

### Modeling Spatially Dependent Cone and Seed Traits by Machine Learning Regression Methods

The number of variables was determined by the rule of ten events per variable (McGarigal et al., 2000; Vittinghoff and McCulloch, 2007, i.e., a maximum of the six most important variables for the 65 *P. strobiformis* stands were included in the models). Regression models, including 5-fold cross-validations, were used to predict the most spatially dependent *P. strobiformis* cone and seed traits for selected important, independent variables in each stand. Six machine learning algorithms were implemented in the “caret” package and function “train” models: (i) linear regression (method = “lm”), (ii) Random Forest (method = “rf”), (iii) Neural Network (method = “nnet”), (iv) Model Averaged Neural Network (method = “avNNet”), (v) Multi-Layer Perceptron (method = “mlpWeightDecay”), and (vi) Bayesian Regularized Neural Networks (method = “brnn”) (Venables and Ripley, 1999; Williams et al., 2018, http://topepo.github.io/caret/index.html) in R (version 3.3.4) (R Development Core Team, 2017). The goodness-of-fit of the regression model was evaluated by using the (pseudo) coefficient of determination (*R*^2^), root of the mean square error (RMSE), and mean squared error.

## Results

### Detecting Spatial Dependence in Morphological Traits by Ordinary Kriging Analysis

**FIGURE 2:**
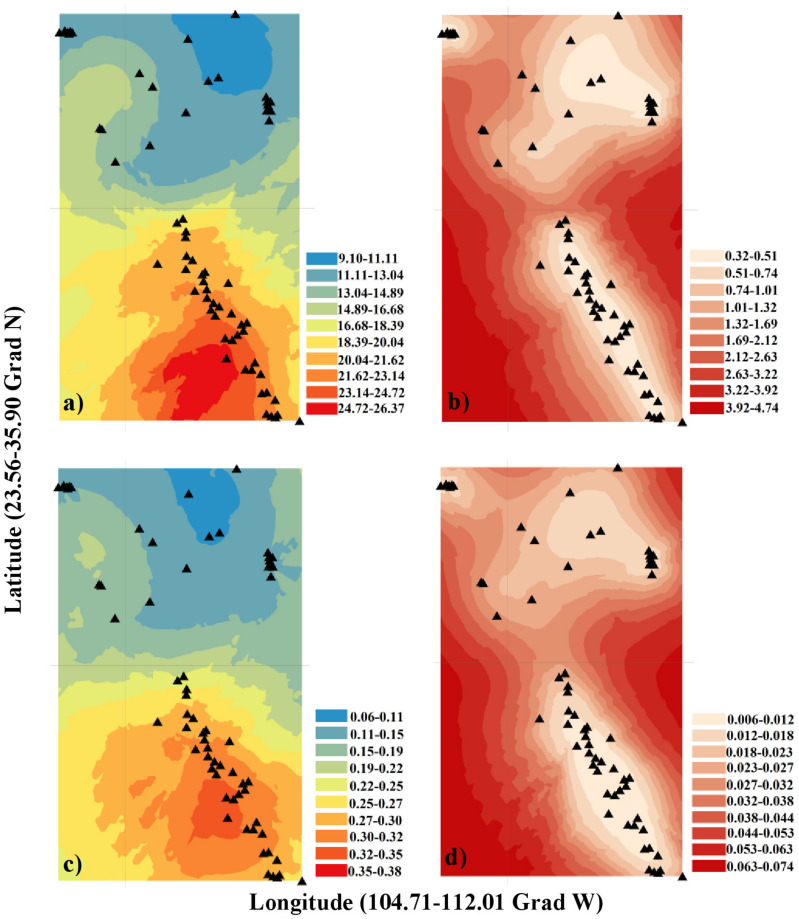
Ordinary kriging model and its standard error (SE) for different morphological traits: **(a)** Cone length (cm), **(b)** SE of cone length (cm), **(c)** Seed weight (g), **(d)** SE of seed weight (g). The statistical software R (version 3.3.4) (R Development Core Team, 2017) and the Interpolation Kriging package (ArcGIS Desktop 10.5, 2016) were used to describe first-order variation in the spatial pattern.

The values of all studied morphological traits increased from the northern (United States) to the southern (Mexican) populations (e.g., cone length and seed weight were larger in southern populations; Figure 2; Supplementary Figure S1). The best kriging model for cone length used Stein’s parameterization (*R_*k*_^2^* = 0.89; *URMSE* = 1.72 cm; Supplementary Table S6). The goodness-of-fit values for the seed weight and scale top angle were slightly lower (*R_*k*_^2^* = 0.75, *URMSE* = 0.04 g; *R_*k*_^2^* = 0.74, *URMSE* = 3.04 mm). In general, the mean cone size, seed weight and scale angle were larger in the southern populations. The worst-performing spatial model was that for scale top width (*R_*k*_^2^* = 0.05). There was a marginally positive relationship between latitude and DBH (*R_*k*_^2^* = 0.06, *p* = 0.033), but there was no association between latitude and tree height (*R_*k*_^2^* = 0.15, *p* = 0.38) (Supplementary Table S6).

### Redundancy Analysis and Variance Partitioning

**FIGURE 3:**
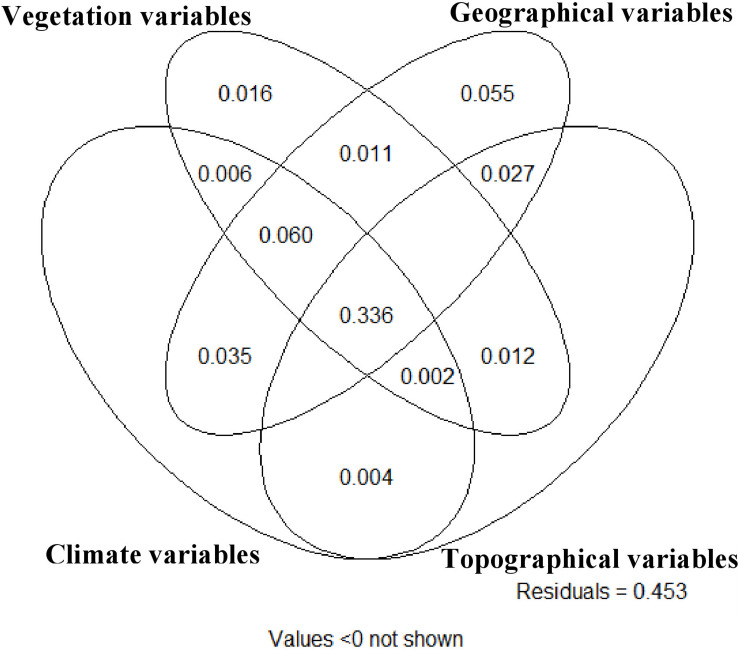
Variance partitioning diagram from partial redundancy analysis among (1) climatic, (2) vegetation, (3) geographical, and (4) topographical variable groups. The total explained variance in morphological characteristics among all sampled individual trees is 54.7% and the numbers in each compartment of the diagram indicate the amount of variance explained by the variable sets overlapping in that compartment.

Collectively, vegetation, spatial, topographic, geographical, and climatic variables explained 54.7% of the total variation in the morphological characteristics among sampled trees (Figure 3). Of the marginal effects of these groups of variables, geographic variables showed the best ability to predict morphological characteristics, accounting for 51.5% of the variance (Supplementary Table S7), followed by vegetation variables (43.8%), climate variables (43.4%) and finally topographic variation (36.9%). There was very high covariation in the explanation among these different sets of variables, with no set explaining more than 6% (set geographic) of the variation in morphological traits independent of the other variable sets. The climate and topographic variable sets had very close to zero (0.004) independent explanatory power. The largest component of explained variance (33.6%) was the four way interaction of all four variable sets, suggesting that there is strong co-variation in environmental, climate and geographical variables in their relationship to morphological characteristics of *P. strobiformis* across its range. The second largest variance component was the three-way interaction between climate, vegetation and geographic variable sets. This shows that in total 39.6% of the variance in morphological traits across the range of southwestern white pine are jointly predicted by simultaneous variation in climate, vegetation and geographic variable sets (Figure 3).

### Modeling Spatially Dependent Cone and Seed Traits Using Machine Learning Methods

For the 65 stands with morphological data, our modeling results indicated that growing season precipitation (GSP) was the most important independent variable for predicting both cone length and seed weight (Supplementary Figures S2, S3). However, the independent variables that together produced the best model of cone length of *P. strobiformis* were the GSP, winter precipitation (WINP), summer precipitation balance (SMRPB), frequency of occurrence of several overstory tree species (*Pseudotsuga menziesii* (Mirbel) Franco, *Pinus cooperi* C.E. Blanco and *Arbutus xalapensis* Kunth), yielding an RMSE of 1.75 cm using the Random Forest (rf) (Table 1; Supplementary Figure S2). The second-best model for cone length (RMSE = 1.83 cm) was produced by the Bayesian Regularized Neural Networks (brnn) and the same independent variables.

**TABLE 1:** Best fit models of cone length (cm) based on 65 *Pinus strobiformis* stands in Mexico and United States.

**Method of variable selection**	**Machine learning algorithm**	**Independent variable**	**RMSE**	**MAE**	**R^2^**
**PLS**	rf	GSP, WINP, SMRPB, *Pseudotsuga menziesii*, *P. cooperi*, *Arbutus xalapensis*	1.755	1.477	0.890
**PLS**	brnn	GSP, WINP, SMRPB, *Pseudotsuga menziesii*, *P. cooperi*, *Arbutus xalapensis*	1.832	1.514	0.867
**ROC**	lm	GSP, *Pseudotsuga menziesii*, MAT, SMRPB, *P. arizonica*, *J. deppeana*	1.939	1.536	0.865
**PLS**	mlpWeightDecay	GSP, WINP, SMRPB, *Pseudotsuga menziesii*, *P. cooperi*, *Arbutus xalapensis*	5.599	4.971	0.191
**KW**	avNNet	GSP, MAT, *Pseudotsuga menziesii*, *P. strobiformis, P. arizonica*, SMRPB	17.852	17.125	0.658
**KW**	nnet	GSP, MAT, *Pseudotsuga menziesii*, *P. strobiformis, P. arizonica*, SMRPB	17.856	17.128	0.621

*PLS = Partial Least Squares, ROC = Receiver Operating Characteristic, KW = Kruskal Wallis, rf = Random Forest, brnn = Bayesian Regularized Neural Networks, lm = linear regression, mlpWeightDecay = Multi-Layer Perceptron, nnet = Neural Network, avNNet = Model Averaged Neural Network, RMSE = Root-mean-square error, MAE = Mean Absolute Error, R^2^ = R squared, GSP = Growing season precipitation, April to September, *Pseudotsuga menziesii* = frequency of occurrence of *Pseudotsuga menziesii* in the neighborhood, SMRPB = Summer precipitation balance: (jul+aug+sep)/(apr+may+jun), *Juniperus deppeana* = frequency of occurrence of *Juniperus deppeana* in the neighborhood, *P. arizonica* = frequency of occurrence of *P. arizonica* in the neighborhood, *P. strobiformis* = frequency of occurrence of *P. strobiformis* in the neighborhood, *P. cooperi* = frequency of occurrence of *P. cooperi* in the neighborhood, *Arbutus xalapensis* = frequency of occurrence of *Arbutus xalapensis, Arctostaphylos pungens* = frequency of occurrence of *Arctostaphylos pungens* MAT = Mean annual temperature (degrees C), WINP = Winter precipitation: (nov+dec+jan+feb).*

In contrast, the GSP, frequency of occurrence of *Pseudotsuga menziesii*, mean annual temperature (MAT), SMRPB, frequency of occurrence of *P. arizonica* Engelm. and *J. deppeana* Steud. in the same site, were the variables that together provided the best prediction of seed weight of *P. strobiformis* [with an RMSE of 0.039 g using linear regression, (lm)]. The second best model of seed weight (RMSE = 0.040 g) was produced using the variables GSP, MAT, frequency of occurrence of *Pseudotsuga menziesii*, frequency of occurrence of *P. strobiformis, P. arizonica* and SMRPB, the brnn approach (Table 2; Supplementary Figures S3). Higher GSP and SMRPB, lower frequency of occurrence of *Pseudotsuga menziesii*, corresponded to longer mean cone length (Supplementary Table S8). Higher GSP, MAT, higher frequency of occurrence of *P. arizonica* and *J. deppeana*, and lower frequency of occurrence of *P. menziesii* and earlier SMRPB were correlated with greater mean seed weights (Supplementary Table S9).

**TABLE 2:** Best fit models of seed weight (g), based on 65 *Pinus strobiformis* stands in Mexico and United States.

**Method of variable selection**	**Machine learning algorithm**	**Independent variables**	**RMSE**	**MAE**	**R^2^**
**ROC**	lm	GSP, *Pseudotsuga menziesii*, MAT, SMRPB, *P. arizonica, J. deppeana*	0.039	0.033	0.798
**KW**	brnn	GSP, MAT, *Pseudotsuga menziesii*, *P. strobiformis, P. arizonica*, SMRPB	0.040	0.031	0.780
**KW**	rf	GSP, MAT, *Pseudotsuga menziesii*, *P. strobiformis, P. arizonica*, SMRPB	0.041	0.033	0.752
**PLS**	avNNet	DD0, *Pseudotsuga menziesii*, *P. arizonica*, *Arctostaphylos pungens, Arbutus xalapensis*, SMRPB	0.043	0.037	0.769
**PLS**	nnet	DD0, *Pseudotsuga menziesii*, *P. arizonica*, *Arctostaphylos pungens, Arbutus xalapensis*, SMRPB	0.048	0.039	0.738
**PLS**	mlpWeightDecay	DD0, *Pseudotsuga menziesii*, *P. arizonica*, *Arctostaphylos pungens, Arbutus xalapensis*, SMRPB	0.075	0.061	0.468

*PLS = Partial Least Squares, ROC = Receiver Operating Characteristic, KW = Kruskal Wallis, rf = Random Forest, brnn = Bayesian Regularized Neural Networks, lm = linear regression, mlpWeightDecay = Multi-Layer Perceptron, nnet = Neural Network, avNNet = Model Averaged Neural Network, RMSE = Root-mean-square error, MAE = Mean Absolute Error, R^2^ = R squared, GSP = Growing season precipitation, April to September, *Pseudotsuga menziesii* = frequency of occurrence of *Pseudotsuga menziesii* in the neighborhood, SMRPB = Summer precipitation balance: (jul+aug+sep)/(apr+may+jun), *Juniperus deppeana* = frequency of occurrence of *Juniperus deppeana* in the neighborhood, *P. arizonica* = frequency of occurrence of *P. arizonica* in the neighborhood, *Arbutus xalapensis* = frequency of occurrence of *Arbutus xalapensis* in the neighborhood, *Arctostaphylos pungens* = frequency of occurrence of *Arctostaphylos pungens* in the neighborhood, MAT = Mean annual temperature (degrees C), *P. strobiformis* = frequency of occurrence of *P. strobiformis* in the neighborhood, DD0 = Degree-days < 0 degrees C (based on mean monthly temperature).*

### Associations of Cone Length and Seed Weight With Tree Dimension and Hybrid Degree

The tree dimensions of DBH, total height and crown length were only marginally and non-significantly correlated with mean cone length (*R*^2^ = 0.03, *p* = 0.15; *R^2^* = 0.02, *p* = 0.25; *R*^2^ = 0.02, *p* = 0.25) and seed weight (*R*^2^ = 0.07, *p* = 0.04; *R*^2^ = 0.003, *p* = 0.67; *R*^2^ = 0.03, *p* = 0.19), respectively. But, there was a moderately negative relationship between hybrid degree and both mean cone length and seed weight (*R*^2^ = 0.30, *p*<0.00001), respectively.

**FIGURE 4:**
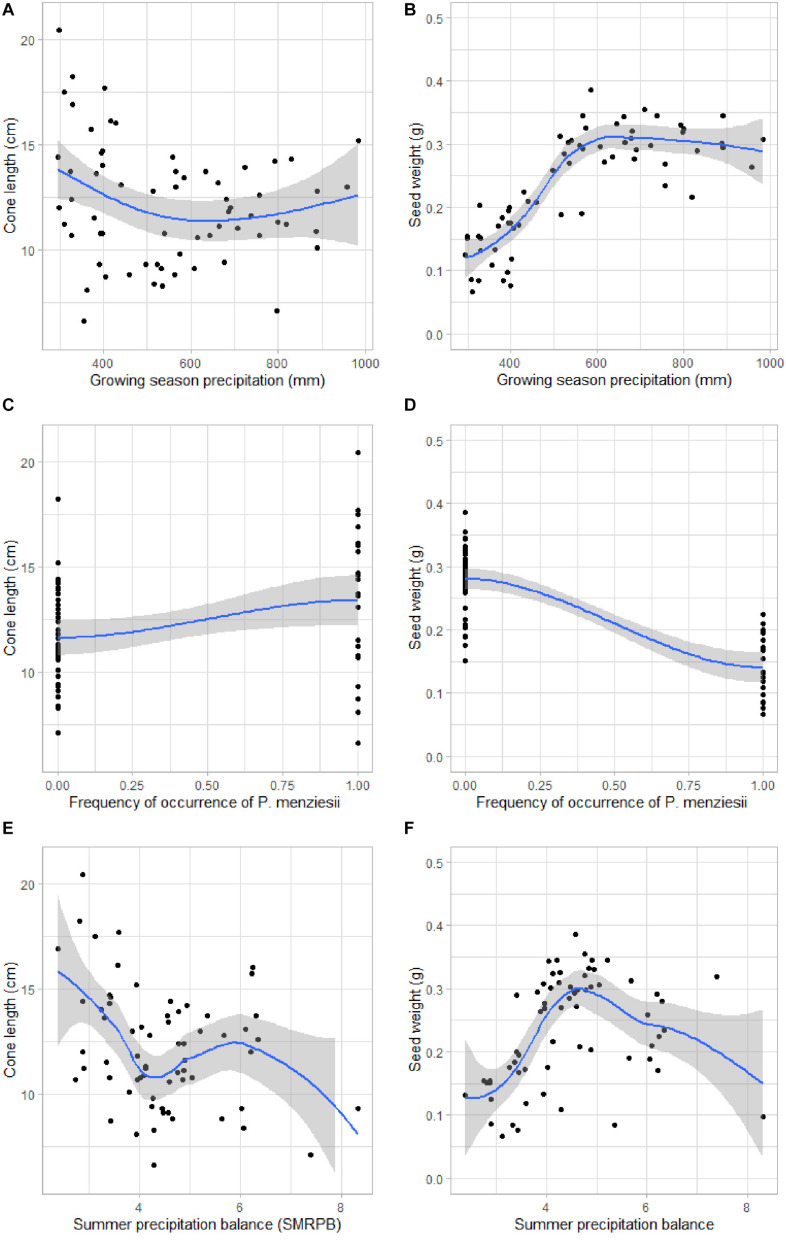
Relationship between the important variables and cone length and seed weight of 65 study sites: **(A)** Cone length (cm) vs. growing season precipitation (mm), **(B)** Seed weight (g) vs. growing season precipitation (mm), **(C)** Cone length (cm) vs. frequency of occurrence of *Pseudotsuga menziesii* in the neighborhood, **(D)** Seed weight (g) vs. frequency of occurrence of *P. menziesii* in the neighborhood, **(E)** Cone length (cm) vs. summer precipitation balance, **(F)** Seed weight (g) vs. summer precipitation balance. The mean (black line) and standard deviation (gray area) is based on the GAM model.

Figure 4 shows the associations between mean cone length and seed weight and the most important variables: growing season precipitation (GSP), frequency of occurrence of *P. menziesii* in the neighborhood, and summer precipitation balance (SMRPB).

## Discussion

### Modeling Spatially Dependent Cone and Seed Traits by Machine Learning Methods

The regression results also illustrated how environmental (GSP, SMRPB, WINP, and MAT) factors influenced the cone length and seed weight of *P. strobiformis*. The largest cones and heaviest seeds were found in more humid and temperate climates, which are located in the southern half of the Mexican Sierra Madre Occidental. This result is in contrast to earlier work in other tree species in the United States by Baker (1972); Schimpf (1977), Sorensen and Miles (1978) and Stromberg and Patten (1990) who found that seeds were larger in drier sites. This difference may reflect the much larger and broader sample of our study, which covered the entire species range and therefore provided a more complete picture of phenotypic variation across the entire distribution than was possible with more limited prior studies. Conversely, Mazer (1989) and Telenius and Torstensson (1991) found no significant association between seed weight and moisture availability; again, those studies were limited by the extent and size of sampling. Leishman and Westoby (1994b) reported that the comparative evidence of an association between large seeds and dry habitats is very limited, despite the general assumption made in the scientific literature. Our study resolves this issue by evaluating a larger, more representative and range-wide sample, and shows strong associations of seed weight and cone length with latitude and climate, with larger seeds and longer cones in the southern, wetter part of the species’ range. However, hybridization with *P. flexilis* also leads to smaller cones and seeds of *P. strobiformis* at its northern border (Menon et al., 2018). Finally, hybridization effects with *P. ayacahuite* should not result in bigger seeds of *P. strobiformis* at the southern distribution border because seeds from *P. ayacahuite* are much smaller. However, hybridization with *P. ayacahuite* may lead to longer cones (Leal-Sáenz et al., 2020).

### Redundancy Analysis and Variance Partitioning

The strong covariation between topographical, geographic, climatic, and vegetation in their relationship to morphological traits suggests that the major variation in the measured morphological traits is associated with the simultaneous and covarying influences of spatial, climatic and topographic factors. The dominant explanatory power of climate, vegetation and geographic variable sets suggest that these factors are particularly important in their relationship to morphological characteristics, while variables in the topographic group have relatively weaker association with morphological traits. The inability of this large empirical sample to statistically separate the effects of climate, vegetation and geographic groups suggests that it is difficult to identify which specific environmental, spatial or climatic factors may be driving observed morphological variation.

The results reported here, however, are useful in showing that a majority of the variability in *P. strobiformis* morphological traits is explainable by climatic, topographical, spatial and vegetation variables and the degree of hybridization with *P. flexilis*. There are strong patterns of morphological variation in this species that are largely explainable by the joint and simultaneous effects of multiple factors. It is not surprising that there is high and inseparable covariation among these factors, as climatic variables are known to change in predictable ways with geographical location (latitude) and topography (elevation). Thus, one hypothesis to explore in common garden experiments is that the variation in morphological traits across the species range are primarily local adaptations to climate, which covaries with elevation and other ecological variables, and that the high apparent relationship between morphology and topography, space and vegetation community are spurious correlations with the actual climatic drivers. A second, alternative, hypothesis could be that competition with other tree species drives the morphological differences across the range, and that the distribution of these competitors is associated with climatic gradients, leading to covariation of morphological traits with climate. Our evidence suggests that cone and seed morphology align with increasing hybridization with *P. flexilis*, resulting in shorter cones and smaller seeds (Steinhoff and Andresen, 1971). Hybridization occurs primarily in the United States populations to the north, creating complex interactions between morphology, climate and genetics (Menon et al., 2018).

Various studies have reported that seeds are often larger under competitive conditions due to the enhanced survival of larger seeds (Salisbury, 1942, 1974; Grime and Jeffrey, 1965; Hutchinson, 1967; Ng, 1978; Foster and Janson, 1985; Mazer, 1989; Leishman and Westoby, 1994a). Egli (2017) argued that variation in seed weight is mainly related to variation in the rate of seed growth during rapid seed filling. Decades of provenance trials in forest trees provide evidence for wide variation in several key ecological traits (Alberto et al., 2013; Lind et al., 2018). For instance, in whitebark pine (*Pinus albicaulis*), Bower and Aitken (2008) suggested that the phenotypic variation is due to genetic and geographic differentiation that reflects the long-term adaptive evolution from the last glacial maximum and from local environmental adaptation. *P. strobiformis* has a large north to south range that results in a gradient of increasing summer precipitation and temperature of the coldest month. The latitudinal climatic gradient along with other abiotic factors also influenced the presence of other tree species around *P. strobiformis*. Therefore, the frequency of occurrence of these species corresponded to the cone length and seed weight as proxy of other abiotic and ecological factors of morphological traits (Zhang et al., 2016, Bañares-de-Dios et al., 2020). For example, we detected *Pseudotsuga menziesii* only in the United States sites, while *Juniperus deppeana, P. arizonica, P. cooperi*, and *A. xalapensis* were found only in the Mexico sites, and in the seed weight model the former species is associated with lower seed weights and the latter species with heavier average seed weights.

Many researchers have argued that variation in cone or seed size within species are closely associated with fitness and, therefore, with adaptive evolution (Roach, 1987; Winn, 1988; Biere, 1991; Platenkamp and Shaw, 1993; Ji et al., 2011). Seed size may also influence seed dispersal (Herrera et al., 1994; Jordano, 1995; Martínez et al., 2007; Shimada et al., 2015). Wang and Ives (2017) reported that seed weight affected almost all choices that rodents made in eating, removing and storing individual seeds. At the level of individual trees, larger seeds have improved probabilities of both predation and effective dispersal. Leslie et al. (2017) also reported that larger seeds were dispersed by animals while smaller seeds were dispersed by the wind and that the morphology of the cones is associated with the size of the seeds. Other studies have reported that latitude, genome size, forest structure, growth form and seed dispersal are related to differences in seed size (Salisbury, 1974; Lord et al., 1997; Leishman et al., 2000; Beaulieu et al., 2007). As the climate continues to shift, the morphological differences between the northern and southern *P. strobiformis* populations may result in differing reforestation patterns and affect management recommendations (Goodrich et al., 2018; Schoettle et al., 2018).

### Common Gardens, Gradient Modeling and Simulation

Experimental common garden studies could separate these influences to some degree, by replicating species combinations across climatic gradients. Common garden experiments are important additionally to quantify the relative degree of phenotypic plasticity and, while they are unlikely to show local adaptation in action, they can show the signature of past local adaptation in the portion of the variance that is not ascribable to phenotypic plasticity. Ultimately, the observed morphological variation is a product of demographic history, local adaptation to climate and environment, phenotypic plasticity, and how these interact in the context of local environmental conditions. The importance of demographic history and genetic drift could be noted in the high explanatory power of geographic variables even though several of them exhibited co-variation with climate and vegetation. To fully resolve the drivers of morphological variation, therefore, replicated and controlled common-garden experiments across the species range, representing combinations of all variable sets (e.g., topography, space, environment and genetics) are essential (Cushman, 2014). These common garden experiments should reciprocally transplant tree families from across the full range, and associate their expressed phenotypic variation with garden specific values of environmental variables, the values of these variables in the location of their maternal trees, and their individual genomic characteristics. Only through replicated and controlled experiments across ecological gradients that associate expressed variation with both environmental and genomic variability can we reliably identify and quantify the drivers of phenotypic variation (Sork et al., 2013).

In practice, however, such common garden studies may have limited practicality given the length of time needed for common garden plots to establish and mature to such a degree that they influence the expression or selection of morphological traits of long lived tree species. In addition, these gardens would have to be large to sufficiently represent forest stand conditions that would reflect competitive dynamics that drive selection or phenotypic plasticity. Finally, given that potentially a large amount of the variation in expressed phenotype is genetically controlled local adaptation, such common gardens would not necessarily show changes in phenotype in time frames feasible for experimentation, given multiple generations of selection are required to observe microevolutionary change in response to selection pressures related to competition or climate. Maintaining a large sample of spatially extensive gardens for many decades is a logistical and financial challenge.

Broad-scale sampling of genomic variation, morphological characteristics, and environmental variables across the species range provides another powerful framework to disentangle space, environment, climate, competition and genetic factors in influencing morphology (e.g., Cushman, 2014). Therefore, it is likely that the most effective way to separate the covarying influences of climate and competition on phenotypic variation will be through large-scale sampling of genomic variation across the population and association of this genomic variation with local environmental, climatic, and community conditions, and partitioning of the variance in morphological traits that are explainable by genomic variation vs. environmental variation. This would show the portion of morphological variation that is genetically correlated, which potentially indicates the degree of local adaptation. Specifically, while sampling standing variation in morphology and genomics across a species range does not enable experimental control necessary to isolate particular drivers with strong inferences, it does allow comparative mensurative designs that can trade space for time, and, critically, sample conditions in situ and at scales in both space and time that are operative and influential on the evolution and community dynamics of trees (McGarigal and Cushman, 2002). Thus we recommend future research combine and couple both broad-scale large-sample analysis of gene-environment gradients across the species range that can describe variation at broad scales and over long timer periods, with targeted, replicated and controlled common garden experiments that can isolate particular drivers of variation at small scales and over short times (Cushman, 2014).

Given the difficulty of reliably isolating drivers and apportioning explanatory variance among them with either observational or common garden experiments on long-lived tree species, we also recommend the use of simulation modeling (e.g., Landguth and Cushman, 2010; Landguth et al., 2017). Specifically, employing an individual-based, spatially explicit eco-evolutionary model (e.g., Landguth et al., 2020) to simulate the interactions of different degrees of gene flow, drift, environmental selection and phenotypic plasticity provides a unique means to explore the potential interactions of these factors and quantify the patterns of genomic and phenotypic variation that can be expected under these interactions (e.g., Cushman, 2015; Cushman and Landguth, 2016).

### Implications for Management and Conservation

The ability of *P. strobiformis* to colonize its expected future range (Shirk et al., 2018) will be influenced by numerous factors, including colonization at the leading edge of the range shift, seed dispersal dynamics, resistance to white pine blister rust, competition with other species, introgression and hybridization (Menon et al., 2020), and genetic adaptation to local climate (Goodrich et al., 2016; Bucholz et al., 2020). Through our results, we have a better understanding of the environmental controls on cone and seed morphology, and we can more adequately evaluate seed provenances and transfer zones and provide better information for assisted migration strategies. Current best practices for seed transfer, such as (a) promoting a tight network of seed stands to prevent greater loss of local genetic variants and structure and (b) using the seeds to establish seedlings within a limited radius from each seed stand/provenance (Hernández-Velasco et al., 2017), are likely to exhibit continued success in the near future. Such practices are essential for current and near-future reforestation programs, including assisted migration or the establishment of new populations in areas that should be appropriate for specific species under expected climate change scenarios (Wehenkel et al., 2017). However, more innovative practices will be required under new climates not conducive to the reproductive success of local populations. This is particularly important given the large projected climate-driven range shift of *P. strobiformis* and predicted large climatic changes within the parts of the range that are likely to remain occupied (Shirk et al., 2018). We recommend a proactive approach, in which reforestation programs incorporate both local and regional seed sources and allow for assisted gene flow (Aitken and Bemmels, 2016). For example, a manager of *P. strobiformis* might include seed sources from the provenance that is most similar to the projected future climate of the planting location (given climate predictions for the species; Shirk et al., 2018) alongside local sources on sites of least aridity (Bucholz et al., 2020). Such a strategy promotes the local genetic structure while providing for adaptation to changing climates and allowing managers flexibility in determining seed sources most suitable given other management considerations (e.g., white pine blister rust). As cone length and seed weight are probably also related to local/regional genetic adaptation (Rawat and Bakshi, 2011), these variables could be included in future climate-based seed transfer guidelines, providing suggestions for average cone lengths and seed weights most appropriate for a given site, including information on degree of hybridization present or desired for a planting site. Given the observed high covariation between climatic, community structure and geographic factors in this study, and the difficulty in separating them statistically or experimentally, there may be no silver bullet for managers to decide on the merits of different planting and assisted migration strategies. Again, given the difficulty of using observational or experimental data to guide these decisions, it may be useful to augment such studies with simulations that can control the interactions of these factors across large ranges of scale in both space and time to quantify the potential roles and influences of each factor in the context and interactions with the others (e.g., Landguth et al., 2017).

### Scope and Limitations

Importantly, these results show that the pattern of morphological variation in *P. strobiformis* is highly predictable in relation to a combination of geographic, floristic, climatic and topographical variables. It is interesting to note that our analysis did not include several environmental variables that are known to strongly predict the distribution of *P. strobiformis* (e.g., soils; Shirk et al., 2018). Thus, it is likely that including a broader set of environmental variables that are limiting to *P. strobiformis* fitness at different scales and in different contexts would increase the amount of variance explained in morphological characteristics across the species range. Additionally, our analysis does not formally integrate observed genomic variation among individual trees in comparison to expressed phenotype across gradients of environmental, climatic and geographic gradients. Future work should therefore focus on collecting these factors simultaneously for a large number of trees across the full extent of the species range and ecological conditions to enable more rigorous evaluation of the degree to which genomic variation can explain observed phenotypic variation, and to what degree it is covarying and thus potentially controlled by selection along environmental gradients. Such studies would also be able to quantify the degree to which admixture with peripatric sister species (e.g., *P. flexilis*) confounds and contributes to observed variation in phenotypic characteristics along geographical and environmental gradients. Another area that would be valuable to integrate into analyses of relationships between phenotypic variation, geography, community structure and environmental gradients would be formal accounting of the influences of phylogeographic and demographic history. Phylogeographic and demographic history create non-stationary and non-equilibrium patterns of genetic structure across populations that are not linearly related to local adaptation or patterns of gene flow (e.g., Dyer et al., 2010). Developing and integrating methods that can account for this therefore is valuable. Again, simulation modeling (Cushman, 2015) may be the most powerful way to account for the interactions of gene flow, drift and selection within varying contexts of phylogeographic and demographic history, given their ability to stipulate and control all the factors that interact in a way that enables simulation experiments to robustly evaluate each factor and its interactions with others.

## Conclusion

We showed that most variation in *P. strobiformis* morphological characteristics is strongly correlated with climatic gradients, suggesting selection for different morphological characteristics under different climatic contexts. However, we could not determine how much of the morphological variation is driven by climate independently of covarying topographical, ecological and spatial factors. In addition, our study does not quantify the degree to which observed morphological variation is genetically controlled, nor how much phenotypic plasticity there is. We advocate for replicated and controlled reciprocal transplant common garden experiments (e.g., Sork et al., 2013; Cushman, 2014) which can enable rigorous separation of the amount of phenotypic variance controlled by genotype, by the environment and by the interaction between genotype and environment. In addition, we suggest coupling such common garden studies to broad-scale gradient modeling of genomic and morphological variation across geographic, climatic and community gradients (Cushman, 2014). Simulation modeling, which can control all the potential factors driving covariation between genomic, phenotypic, geographical, and environmental factors at scales in space and time relevant to population responses are likely to be critical to rigorously quantify these relationships and untangle them. Ultimately a combination of common garden, gradient modeling and simulation studies provides the best means for advancing the important and challenge task of understanding and predicting eco-evolutionary dynamics across broad populations in complex and dynamic environments (Cushman, 2014). The results presented here, however, are useful and important in showing spatial range-wide patterns of phenotypic variation that are strongly associated with environmental gradients, and that a large portion of this variation is also associated with the joint effects of climate, topography, latitude and regional vegetation community composition.

The combination of variance partitioning and machine learning algorithms implemented here provides a clear demonstration of both the relative importance and independent effects of different climatic, geographic and environmental factors in driving morphological variation in *P. strobiformis*, but also identify the main patterns of this variation and the variables that are most strongly associated with them. We find that cone length, seed weight and cone morphology are strongly related to temperature and precipitation and increase in warmer and wetter parts of the species range. These results show that spatial modeling across a species range can yield accurate predictions of morphological traits as a function of environmental gradients. These models predict the considerable differences in geographical, topographical, climate, adaptive and morphological variables in the species range and may help to distinguish the actual seed provenance of *P. strobiformis*.

## Data Availability Statement

The raw data supporting the conclusions of this article will be made available by the authors, without undue reservation.

## Author Contributions

CW and KW conceived and designed the experiments. KW, AL-S, and CW conducted sampling. AL-S, CW, and SC analyzed the data. ALS and CW prepared figures and tables and contributed reagents, materials, and analysis tools. AL-S, LF-R, and AE performed the experiments. CW, SC, KW, and AL-S wrote the manuscript. KW, CW, MM, SC, AE, LF-R, JH-D, CL-S, and JM-G reviewed drafts of the manuscript. All authors contributed to the article and approved the submitted version.

## Conflict of Interest

The authors declare that the research was conducted in the absence of any commercial or financial relationships that could be construed as a potential conflict of interest.

## Footnotes

1 http://forest.moscowfsl.wsu.edu/climate/
