# Supplementary material for: Morphological Differences in Pinus strobiformis Across Latitudinal and Elevational Gradients
Source: Front Plant Sci. 2020 Oct 22;11:559697. doi: 10.3389/fpls.2020.559697 (PMC7642095; doi:10.3389/fpls.2020.559697)
Supplement: Supplementary file 1 [file Data_Sheet_1.docx]

Supplementary Material

**Supplementary Table 1 |** Sample tree attributes of morphological characteristics, dasometric traits and environmental trait determined in 65 *Pinus* *strobiformis* stands in Mexico and United States. DBH = Diameter at breast height, Height = tree height, HBLC = height of lowest live branch, Crown length = Distance from the last green branch to the total height of the tree, Hybrid degree = proportion of *P. strobiformis* to *P. flexilis* per stand. SD = standard deviation.

| Variable | Min | Max | Mean | SD |
| --- | --- | --- | --- | --- |
| Cone length (cm) | 9.1 | 26.4 | 18.1 | 5.1 |
| Cone width (cm) | 4.3 | 9.7 | 7.4 | 1.4 |
| Cone angle (°) | 7 | 36 | 18 | 7 |
| Scale top angle (°) | 2.6 | 23.6 | 13.9 | 6.1 |
| Scale top length (mm) | 22.3 | 42.0 | 32 | 5 |
| Scale top width (mm) | 15.4 | 29.8 | 23.6 | 3.3 |
| Scale bottom angle (°) | 2 | 22.4 | 13.4 | 5.6 |
| Scale bottom length (mm) | 22.8 | 40.7 | 32 | 4.7 |
| Scale bottom width (mm) | 14.1 | 27.6 | 22.2 | 3.1 |
| Seed weight (g) | 0.067 | 0.386 | 0.235 | 0.086 |
| Dasometric trait |  |  |  |  |
| DBH (cm) | 20.7 | 75.2 | 40 | 11 |
| Height (m) | 7.2 | 23 | 16.5 | 3.5 |
| HBLC (m) | 0 | 9.9 | 4.3 | 2.3 |
| Crown length (m) | 6.6 | 20.4 | 12.2 | 2.8 |
| Environmental trait |  |  |  |  |
| Hybrid degree | 0 | 0.40 | 0.04 | 0.12 |

**Supplementary Table 2 |** Sample tree attributes of site characteristics determined in 65 *Pinus* *strobiformis* stands in Mexico and United States. Aspect = geographical aspect, Lat = Latitude, Long = Longitude, WPBR = Frequency of occurrence of white pine blister rust in the neighbourhood.

| Variable | Min | Max | Mean | SD |
| --- | --- | --- | --- | --- |
| Slope (%) | 2 | 68 | 29 | 14 |
| Aspect (°) | 3 | 316 | 88 | 92 |
| Lat N (°) | 23.562 | 35.904 | 29.429 | 3.906 |
| Long W (°) | 104.715 | 112.010 | 107.522 | 1.915 |
| Elevation (m) | 2,036 | 3,127 | 2,527 | 235 |
| WPBR | 0.00 | 0.80 | 0.03 | 0.13 |

**Supplementary Table 3 |** Sample tree attributes of climates characteristics determined in 65 stands of Mexico and United States (University of Idaho, USA (<http://forest.moscowfsl.wsu.edu/climate/>)). SD = standard deviation.

| Variable | Min | Max | Mean | SD |
| --- | --- | --- | --- | --- |
| Mean annual temperature (degrees C) (MAT) | 3.1 | 12.7 | 9.3 | 2.6 |
| Mean annual precipitation (mm) (MAP) | 526 | 1,359 | 819 | 212 |
| Growing season precipitation, April to September (GSP) | 295 | 983 | 552 | 189 |
| Mean temperature (degrees C) in the coldest month (MTCM) | -5.8 | 7.2 | 2.4 | 3.8 |
| Mean minimum temperature (degrees C) in the coldest month (MMIN) | -12.8 | -1.3 | -6.1 | 2.9 |
| Mean temperature (degrees C) in the warmest month (MTWM) | 13.4 | 20 | 16.3 | 1.6 |
| Mean maximum temperature in the warmest month (MMAX) | 21.2 | 28.2 | 24.8 | 2 |
| Julian date of the last freezing date of spring (SDAY) | 122 | 183 | 155 | 15 |
| Julian date of the first freezing date of autumn (FDAY) | 240 | 300 | 274 | 16 |
| Length of the frost-free period (days) (FFP) | 55 | 184 | 124 | 31 |
| Degree-days >5 degrees C (based on mean monthly temperature) (DD5) | 887 | 2,918 | 2,029 | 519 |
| Degree-days >5 degrees C accumulating within the frost-free period (GSDD5) | 455 | 2,166 | 1,251 | 406 |
| Julian date the sum of degree-days >5 degrees C reaches 100 (D100) | 35 | 158 | 82 | 36 |
| Degree-days <0 degrees C (based on mean monthly temperature) (DD0) | 0 | 843 | 173 | 253 |
| Degree-days <0 degrees C (based on mean minimum monthly temperature) (MMINDD0) | 439 | 2,307 | 1,082 | 500 |
| Summer precipitation balance: (jul+aug+sep)/(apr+may+jun) (SMRPB) | 2.39 | 8.31 | 4.45 | 1.17 |
| ((Depreciated) Summer/Spring precipitation balance: (jul+aug)/(apr+may) (SMRSPRPB) | 2.58 | 21.07 | 9.99 | 4.47 |
| Spring precipitation: (apr+may) (SPRP) | 24 | 70 | 38 | 12 |
| Summer precipitation: (jul+aug) (SMRP) | 165 | 569 | 336 | 118 |
| Winter precipitation: (nov+dec+jan+feb) (WINP) | 82 | 333 | 183 | 53 |

**Supplementary Table 4 |** Frequency of occurrence of tree species taken from 65 *Pinus strobformis* stands in Mexico and United States. SD = standard deviation.

| *Variable = Frequency of occurrence of: | Min | Max | Mean | SD |
| --- | --- | --- | --- | --- |
| *Pseudotsuga menziesii* | 0 | 1 | 0.32 | 0.47 |
| *Pinus ponderosa* | 0 | 1 | 0.31 | 0.47 |
| *Pinus arizonica* | 0 | 1 | 0.34 | 0.48 |
| *Pinus strobiformis* | 0 | 1 | 0.55 | 0.50 |
| *Pinus durangensis* | 0 | 1 | 0.22 | 0.41 |
| *Quercus sideroxyla* | 0 | 1 | 0.20 | 0.40 |
| *Populus tremuloides* | 0 | 1 | 0.09 | 0.29 |
| *Pinus cooperi* | 0 | 1 | 0.12 | 0.33 |
| *Pinus leiophylla* | 0 | 1 | 0.12 | 0.33 |
| *Pinus lumholtzii* | 0 | 1 | 0.11 | 0.31 |
| *Pinus teocote* | 0 | 1 | 0.09 | 0.29 |
| *Pinus engelmannii* | 0 | 1 | 0.08 | 0.27 |
| *Abies concolor* | 0 | 1 | 0.05 | 0.21 |
| *Quercus fulva* | 0 | 1 | 0.06 | 0.24 |
| *Picea pungens* | 0 | 1 | 0.02 | 0.12 |
| *Quercus gambelii* | 0 | 1 | 0.05 | 0.21 |
| *Cupressus spp.* | 0 | 1 | 0.03 | 0.17 |
| *Pinus pseudostrobus* | 0 | 1 | 0.02 | 0.12 |
| *Ribes spp.* | 0 | 1 | 0.28 | 0.45 |
| Regeneration of *P. strobiformis* | 0 | 1 | 0.85 | 0.36 |
| *Juniperus deppeana* | 0 | 1 | 0.28 | 0.45 |

*The frequency of occurrence in the neighbourhood

**Supplementary Table 5 |** Sample species shrubs attributes taken from 65 stands of Mexico and United States. SD = standard deviation.

| *Variable = Frequency of occurrence of: | Min | Max | Mean | SD |
| --- | --- | --- | --- | --- |
| *Filicopsida* | 0 | 1 | 0.08 | 0.27 |
| *Robinia neomexicana* | 0 | 1 | 0.02 | 0.12 |
| *Castilleja angustifolia* | 0 | 1 | 0.02 | 0.12 |
| *Quercus berberidifolia* | 0 | 1 | 0.02 | 0.12 |
| *Arbutus xalapensis* | 0 | 1 | 0.23 | 0.42 |
| *Holodiscus discolor* | 0 | 1 | 0.02 | 0.12 |
| *Berberis aquifolium* | 0 | 0.6 | 0.01 | 0.08 |
| *Arctostaphylos pungens* | 0 | 1 | 0.35 | 0.48 |

*The frequency of occurrence in the neighbourhood

**Supplementary Table 6 |** Results of kriging models of morphological traits. MAE =Mean absolute error, MSE = Mean square error, URMSE = unbiased root mean square errors, Model = model used for the semivariance: Ste (Matern, M. Stein's parameterization), Exp (Exponential), Sph (Spherical), *R_k_^2^* = corrected coefficient of determination. HBLC = height of lowest live branch, Tree height= total height of the tree, DBH= Diameter at breast height.

| Morphological trait | MAE | MSE | URMSE | Model | *R_k_^2^* |
| --- | --- | --- | --- | --- | --- |
| Cone length (cm) | 1.49 | 2.96 | 1.72 | Ste | 0.89 |
| Seed weight (g) | 0.03 | <0.01 | 0.04 | Exp | 0.75 |
| Scale top angle (°) | 2.59 | 9.23 | 3.04 | Ste | 0.74 |
| Scale bottom angle (°) | 2.40 | 8.19 | 2.86 | Sph | 0.74 |
| Scale top length (mm) | 2.47 | 9.63 | 3.10 | Ste | 0.61 |
| Scale bottom length (°) | 2.57 | 9.76 | 3.12 | Ste | 0.56 |
| Cone width (cm) | 0.88 | 1.24 | 1.11 | Exp | 0.43 |
| Scale bottom width (mm) | 1.89 | 6.04 | 2.46 | Ste | 0.41 |
| Cone angle (°) | 4.11 | 31.33 | 5.60 | Ste | 0.22 |
| Scale top width (mm) | 2.57 | 10.36 | 3.22 | Gau | 0.05 |
| Dasometric trait |  |  |  |  |  |
| HBLC (m) | 1.47 | 3.73 | 1.93 | Exp | 0.27 |
| Tree height (m) | 3.10 | 14.89 | 3.86 | Sph | 0.15 |
| DBH (cm) | 7.98 | 110.50 | 10.51 | Exp | 0.06 |
| Crown length (m) | 2.26 | 7.41 | 2.72 | Ste | 0.05 |

**Supplementary Table 7** | Results of RDA analysis and variance partitioning.

| **Component** | **Description of Component** | **Variance Explained by Component** |
| --- | --- | --- |
| Marginal x1 | Climate Total | 0.434 |
| Marginal x2 | Vegetation Total | 0.438 |
| Marginal x3 | Geographic- Total | 0.515 |
| Marginal x4 | Topographic Total | 0.369 |
| x1 | Climate Only | 0 |
| x2 | Vegetation Only | 0.016 |
| x3 | Geographic Only | 0.055 |
| x4 | Topographic Only | 0 |
| x1x2 | Climate and Vegetation Only | 0.006 |
| x1x3 | Climate and Geographic Only | 0.035 |
| x1x4 | Climate and Topographic Only | 0.004 |
| x2x3 | Vegetation and Geographic Only | 0.011 |
| x2x4 | Vegetation and Topographic Only | 0.012 |
| x3x4 | Geographic and Topographic Only | 0.027 |
| x1x2x3 | Climate, Vegetation and Geographic Only | 0.060 |
| x1x2x4 | Climate, Vegetation and Topographic Only | 0.002 |
| x1x3x4 | Climate, Geographic and Topographic Only | 0 |
| x2x3x4 | Vegetation, Geographic and Topographic Only | 0 |
| x1x2x3x4 | All four Jointly | 0.336 |

**Supplementary Table 8 |** Parameters for the best linear regression model of cone length based on 65 *Pinus strobiformis* stands (see also Table 1).

| (Intercept) | 8.8730 |
| --- | --- |
| GSP | 0.0127 |
| *Pseudotsuga menziesii* | -2.9616 |
| MAT | 0.0734 |
| SMRPB | 0.2673 |
| *Pinus arizonica* | 2.5923 |
| *Juniperus deppeana* | 1.6268 |

Note: GSP = Growing season precipitation, April to September, *Pseudotsuga menziesii* = frequency of occurrence of *Pseudotsuga menziesii* in the neighbourhood*,* SMRPB = Summer precipitation balance: (jul+aug+sep)/(apr+may+jun), *Juniperus deppeana* = frequency of occurrence of *Juniperus deppeana* in the neighbourhood*, Pinus arizonica =* frequency of occurrence of *P. arizonica* in the neighbourhood*,* MAT = Mean annual temperature (degrees C)*.*

**Supplementary Table 9 |** Parameters of the best linear regression model of seed weight based on 65 *Pinus strobiformis* stands (see also Table 2).

| (Intercept) | 0.1052 |
| --- | --- |
| GSP | 0.0001 |
| *Pseudotsuga menziesii* | -0.0496 |
| MAT | 0.0052 |
| SMRPB | -0.0029 |
| *Pinus arizonica* | 0.0489 |
| *Juniperus deppeana* | 0.0212 |

Note: GSP = Growing season precipitation, April to September, *Pseudotsuga menziesii* = frequency of occurrence of *Pseudotsuga menziesii* in the neighbourhood*,* SMRPB = Summer precipitation balance: (jul+aug+sep)/(apr+may+jun), *Juniperus deppeana* = frequency of occurrence of *Juniperus deppeana* in the neighbourhood, *Pinus arizonica =* frequency of occurrence of *P. arizonica* in the neighbourhood, MAT = Mean annual temperature (degrees C).

**FIGURES**


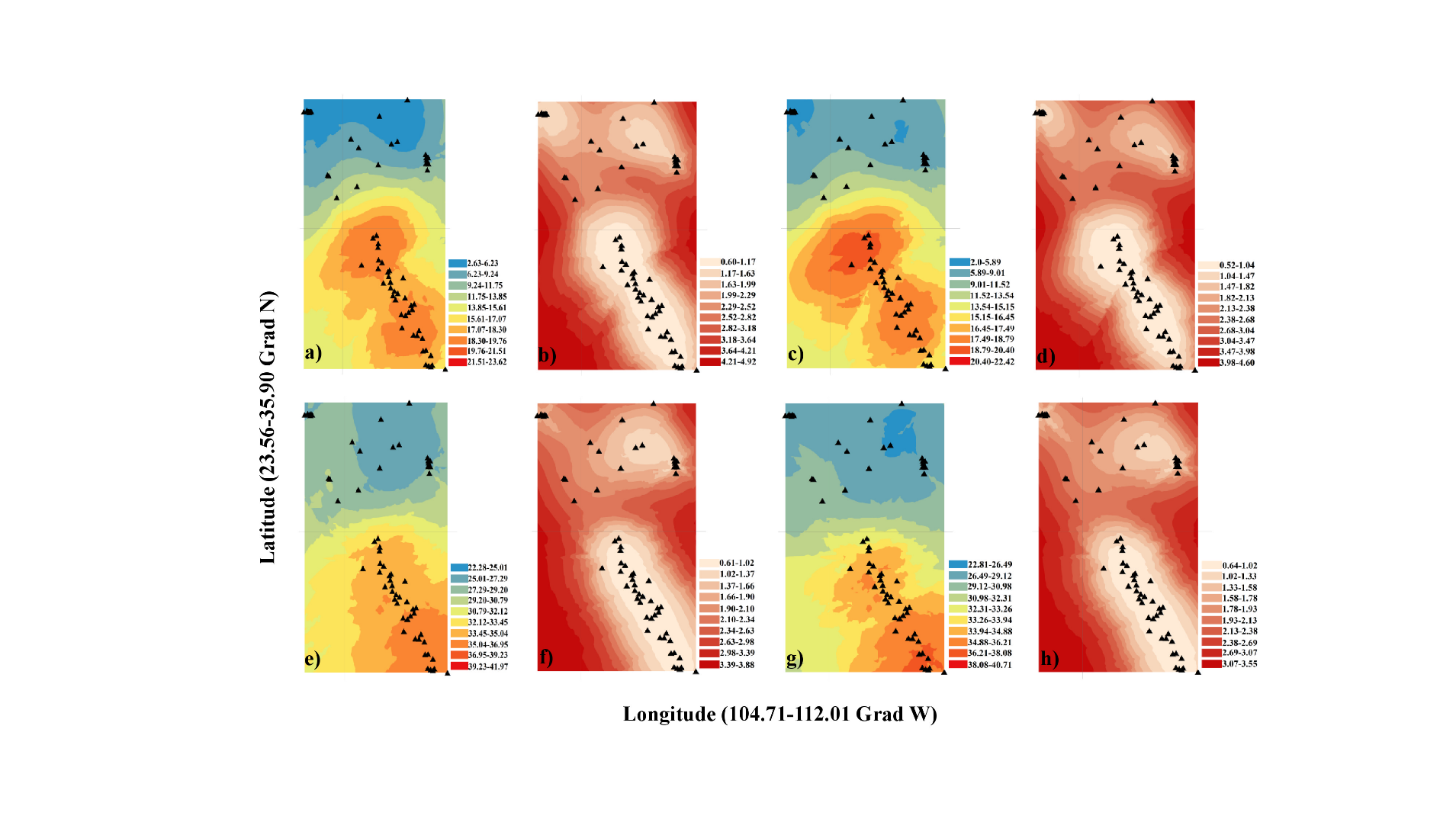


**Supplementary Figure 1 |** Ordinary kriging model and its standard error (SE) for different morphological traits: a) Scale top angle (°), b) SE of scale top angle (°), c) Scale bottom angle (°), d) SE of scale bottom angle (°), e) Scale top length (mm), f) SE of scale top length (mm), g) Scale bottom length (mm), h) SE of scale bottom length (mm).

**Supplementary Figure 2 |** Important variables for the model of cone length based on 65 *Pinus strobiformis* stands selected by PLS (Partial Least Squares) and the Random Forest algorithm; GSP = Growing season precipitation, April to September, SMRP = Summer precipitation: (jul+aug), DD0 = Degree-days <0 degrees C (based on mean monthly temperature), D100 = Julian date the sum of degree-days >5 degrees C reaches 100, MAP = Mean annual precipitation (mm), Aspect = geographical aspect, SMRSPRPB = Summer/Spring precipitation balance: (jul+aug)/(apr+may), MTCM = Mean temperature (degrees C) in the coldest month, WINP = Winter precipitation: (nov+dec+jan+feb). SMRPB = Summer precipitation balance: (jul+aug+sep)/(apr+may+jun), Pm = frequency of occurrence of *Pseudotsuga menziesii* in the neighbourhood*,* Pc = frequency of occurrence of *Pinus cooperi* in the neighbourhood, SPRP = Spring precipitation: (apr+may), Ax = frequency of occurrence of *Arbutus xalapensis* in the neighbourhood, MMAX = Mean maximum temperature in the warmest month, MTWM = Mean temperature (degrees C) in the warmest month, Jd = frequency of occurrence of *Juniperus deppeana* in the neighbourhood, Slope = slope position (%), FFP = Length of the frost-free period (days), Pa *=* frequency of occurrence of *Pinus arizonica* in the neighbourhood*.*

**Supplementary Figure 3 |** Important variables for the model of seed weight based on 65 *Pinus strobifomis* stands and selected by ROC (Receiver Operating Characteristic) and linear regression; GSP = Growing season precipitation, April to September, SMRP = Summer precipitation: (jul+aug), MTCM = Mean temperature (degrees C) in the coldest month, DD0 = Degree-days <0 degrees C (based on mean monthly temperature), D100 = Julian date the sum of degree-days >5 degrees C reaches 100, SMRSPRPB = Summer/Spring precipitation balance: (jul+aug)/(apr+may), Aspect = geographical aspect, MAP = Mean annual precipitation (mm), Pm = frequency of occurrence of *Pseudotsuga menziesii* in the neighbourhood*,* MAT = Mean annual temperature (degrees C), MMIN = Mean minimum temperature (degrees C) in the coldest month, SMRPB = Summer precipitation balance: (jul+aug+sep)/(apr+may+jun), Pp = frequency of occurrence of *Pinus ponderosa* in the neighbourhood, SPRP = Spring precipitation: (apr+may), DD5 = Degree-days >5 degrees C (based on mean monthly temperature), MMINDD0 = Degree-days <0 degrees C (based on mean minimum monthly temperature), Pa *=* frequency of occurrence of *Pinus arizonica* in the neighbourhood*,* Jd = frequency of occurrence of *Juniperus deppeana* in the neighbourhood, Ap *=* frequency of occurrence of *Arctostaphylos pungens* in the neighbourhood, FDAY = Julian date of the first freezing date of autumn.
