# Supplementary figures and images for: Morphological Differences in Pinus strobiformis Across Latitudinal and Elevational Gradients
Source: Front Plant Sci. 2020 Oct 22;11:559697. doi: 10.3389/fpls.2020.559697 (PMC7642095; doi:10.3389/fpls.2020.559697)

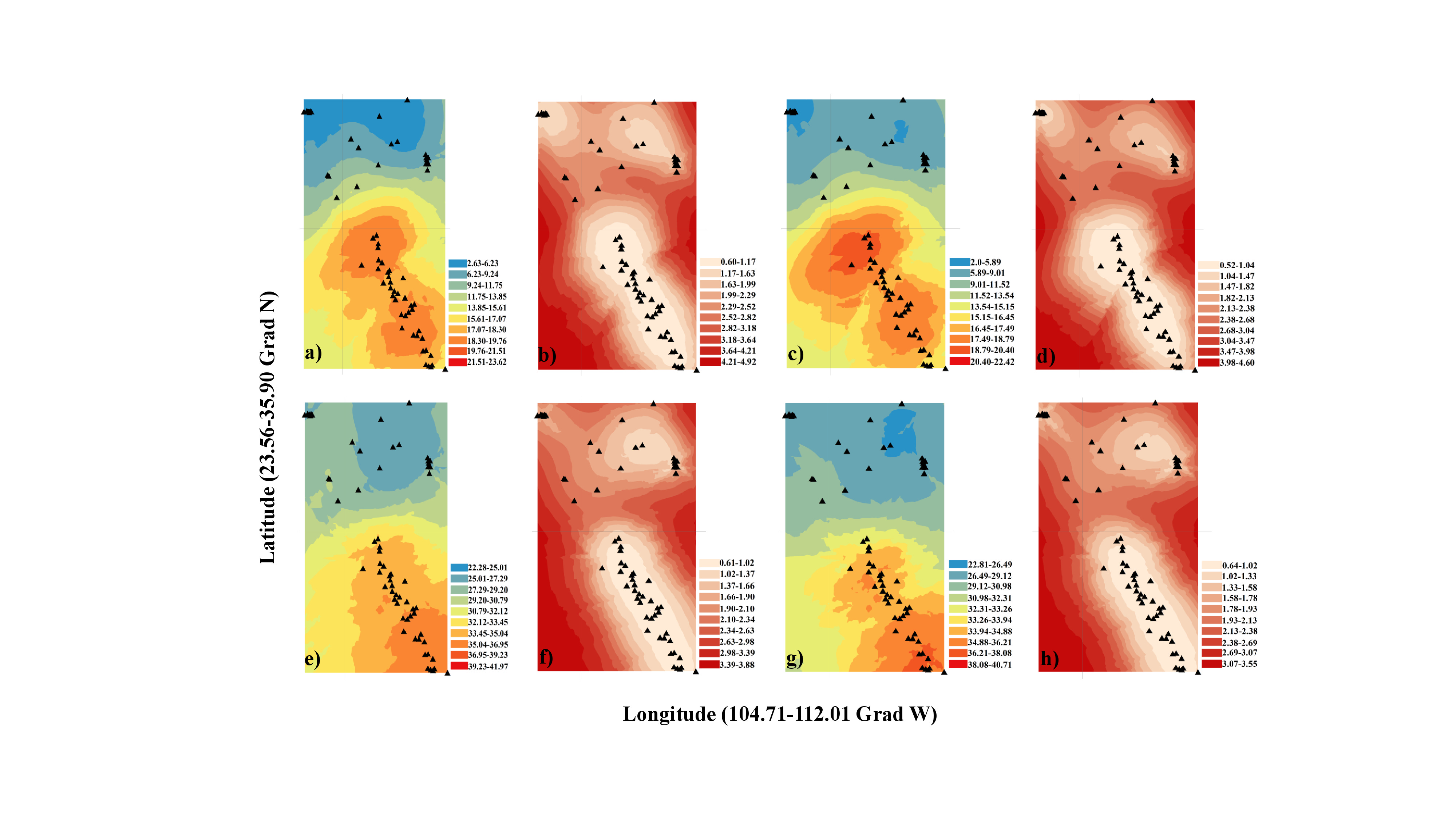

Supplement: Supplementary file 3 [file Image_1.TIF]

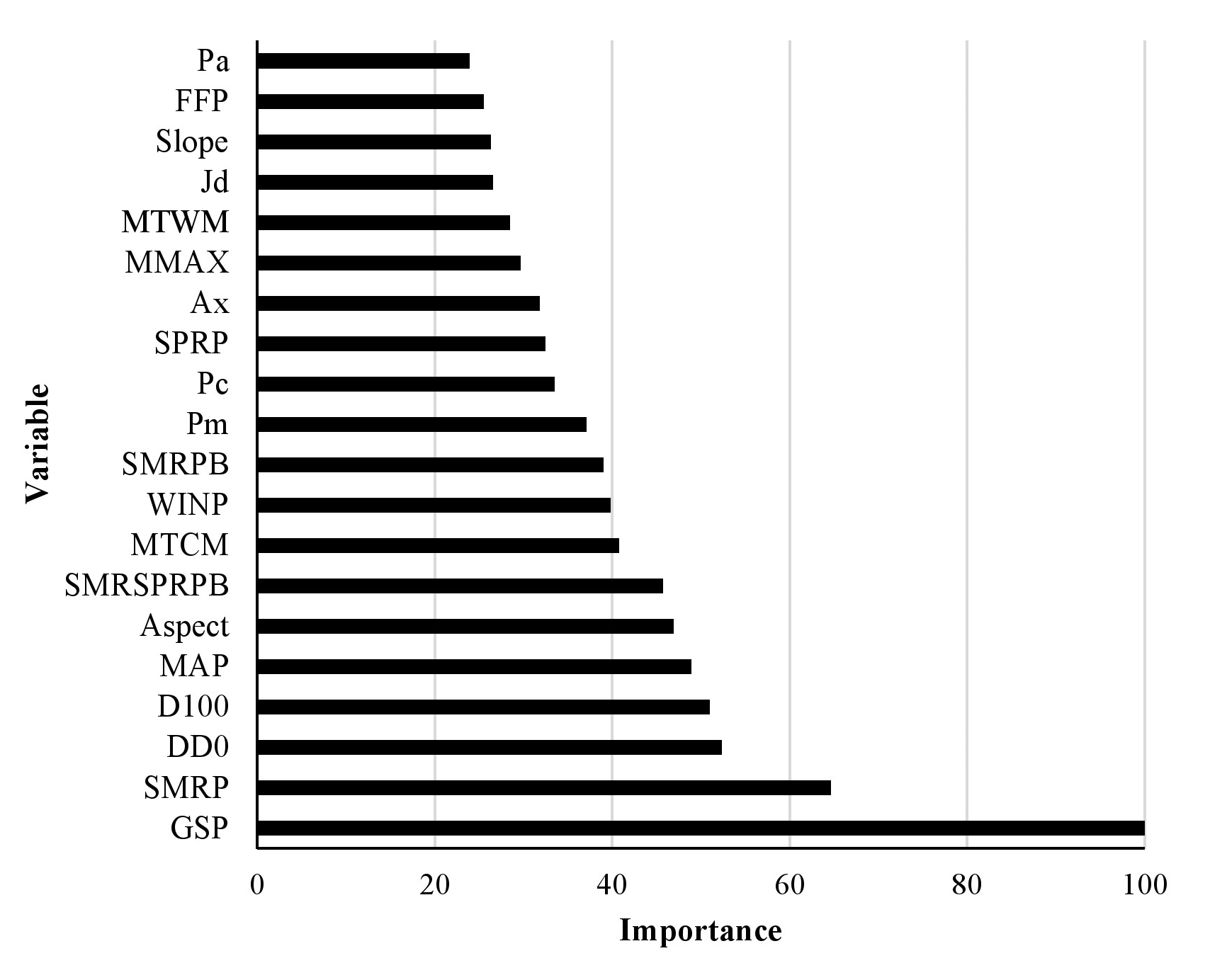

Supplement: Supplementary file 4 [file Image_2.TIF]

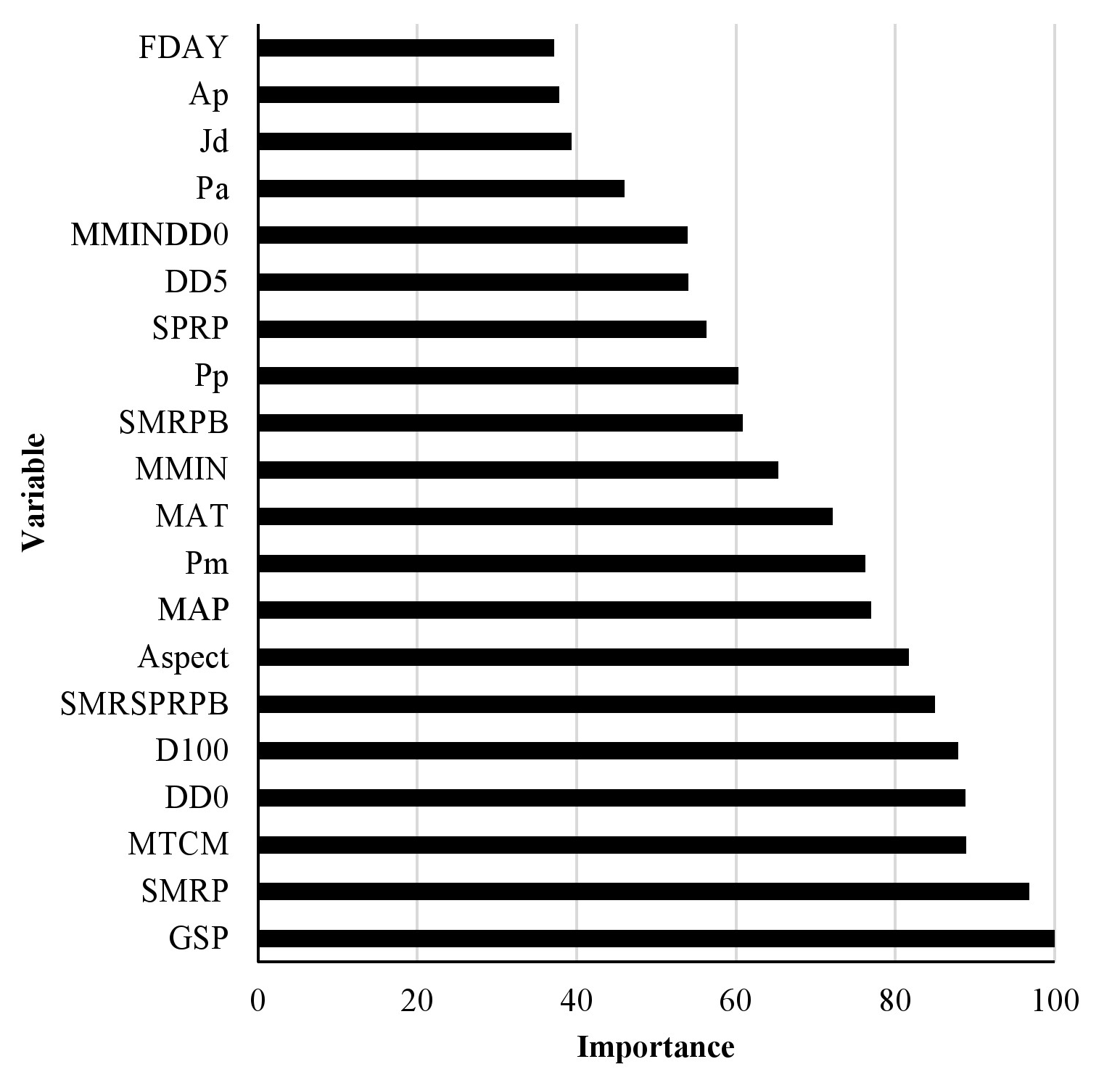

Supplement: Supplementary file 5 [file Image_3.TIF]
